# Prior stress and vasopressin promote corticotropin-releasing factor inhibition of serotonin release in the central nucleus of the amygdala

**DOI:** 10.3389/fnbeh.2023.1148292

**Published:** 2023-03-30

**Authors:** Patrick J. Ronan, Wayne J. Korzan, Philip L. Johnson, Christopher A. Lowry, Kenneth J. Renner, Cliff H. Summers

**Affiliations:** ^1^Research Service, Sioux Falls VA Health Care System, Sioux Falls, SD, United States; ^2^Department of Psychiatry, Sanford School of Medicine, University of South Dakota, Sioux Falls, SD, United States; ^3^Laboratory for Clinical and Translational Research in Psychiatry, Department of Veterans Affairs Medical Center, Denver, CO, United States; ^4^Neuroscience Group, Division of Basic Biomedical Sciences, Sanford School of Medicine, University of South Dakota, Vermillion, SD, United States; ^5^Department of Biological and Environmental Sciences, The University of West Alabama, Livingston, AL, United States; ^6^Department of Biology, University of South Dakota, Vermillion, SD, United States; ^7^Department of Integrative Physiology, University of Colorado, Boulder, Boulder, CO, United States

**Keywords:** CRF, AVP, restraint stress, 5-HT, CeA, affective disorders

## Abstract

Corticotropin-releasing factor (CRF) is essential for coordinating endocrine and neural responses to stress, frequently facilitated by vasopressin (AVP). Previous work has linked CRF hypersecretion, binding site changes, and dysfunctional serotonergic transmission with anxiety and affective disorders, including clinical depression. Crucially, CRF can alter serotonergic activity. In the dorsal raphé nucleus and serotonin (5-HT) terminal regions, CRF effects can be stimulatory or inhibitory, depending on the dose, site, and receptor type activated. Prior stress alters CRF neurotransmission and CRF-mediated behaviors. Lateral, medial, and ventral subdivisions of the central nucleus of the amygdala (CeA) produce CRF and coordinate stress responsiveness. The purpose of these experiments was to determine the effect of intracerebroventricular (icv) administration of CRF and AVP on extracellular 5-HT as an index of 5-HT release in the CeA, using *in vivo* microdialysis in freely moving rats and high performance liquid chromatography (HPLC) analysis. We also examined the effect of prior stress (1 h restraint, 24 h prior) on CRF- and AVP-mediated release of 5-HT within the CeA. Our results show that icv CRF infusion in unstressed animals had no effect on 5-HT release in the CeA. Conversely, in rats with prior stress, CRF caused a profound dose-dependent decrease in 5-HT release within the CeA. This effect was long-lasting (240 min) and was mimicked by CRF plus AVP infusion without stress. Thus, prior stress and AVP functionally alter CRF-mediated neurotransmission and sensitize CRF-induced inhibition of 5-HT release, suggesting that this is a potential mechanism underlying stress-induced affective reactivity in humans.

## Introduction

The central nucleus of the amygdala (CeA) plays a major role in neuroendocrine, autonomic and behavioral responses to stress (Chaouloff, 1993; Gray, 1993; Herman and Cullinan, 1997; Ronan and Summers, 2011; Callahan et al., 2013; Gilpin et al., 2014; van den Burg and Stoop, 2019). Lesions of the CeA diminish hypothalamic-pituitary-adrenal (HPA) activation in response to immobilization stress (Beaulieu et al., 1987), and also produce deficits in the learning about stressors (Maren, 1998). Knocking down corticotropin-releasing factor (CRF) expression in the CeA, reduces, whereas overexpression of CeA CRF enhances, anxious behavior (Regev et al., 2012). Furthermore, excitation of the CeA, or activation of serotonin (5-HT) receptors within the CeA, activate the HPA stress axis (Feldman and Weidenfeld, 1998; Feldman et al., 1998, 2000). Serotonergic activity within the CeA during stress (Adell et al., 1997; Hayley et al., 2001) derives from innervation by 5-HT cell groups primarily in the dorsal raphé nucleus (dRN), but also from minor projections from the median raphé nucleus (mRN) (Vertes, 1991; Hale et al., 2008; Johnson et al., 2015), that are activated during stress (Chaouloff, 1993; Adell et al., 1997; Chaouloff et al., 1999; Grahn et al., 1999; Roche et al., 2003; Summers et al., 2003). Coordinated central CRF circuitry, including CeA (Gray, 1993; Swanson and Petrovich, 1998; Makino et al., 2002; Ronan and Summers, 2011), acts on the dRN (Lowry et al., 2000; Summers et al., 2003; Lukkes et al., 2011) and results in serotonergic responses in limbic brain regions (Forster et al., 2006, 2008), including the amygdala, and specifically the CeA (Mo et al., 2008). In rodents, intracerebroventricular (icv) administration of CRF facilitates a variety of behavioral and physiological responses that mimic those seen during exposure to acute stressors (Dunn and Berridge, 1990; Koob et al., 1993; Holsboer, 1999), which can be reversed by intra-CeA injection of CRF receptor antagonists (Koob et al., 1993; Rassnick et al., 1993). What is more, the neuropeptide arginine vasopressin (AVP) also innervates the CeA and dRN and influences 5-HT activity along with anxiety (Rood and Beck, 2014; Hernandez et al., 2016). Therefore, it appears that AVP, CRF and 5-HT in the dRN and CeA are important modulators of stress behavior.

Several lines of evidence suggest a role for central CRF in the regulation of neural 5-HT at the level of the raphé nuclei, as well as 5-HT regulation of CRF (Marcinkiewcz et al., 2016). For example, dRN and mRN neurons express mRNA and protein for both CRF_1_ and CRF_2_ receptors (Chalmers et al., 1995, 1996; Van Pett et al., 2000; Roche et al., 2003). Immunoreactive CRF terminals and fibers are located within the dRN and mRN (Swanson et al., 1983; Sakanaka et al., 1987; Kirby et al., 2000; Roche et al., 2003) derived, in part, from CeA CRF neurons (Swanson et al., 1983; Gray, 1993), and in synaptic contact with 5-HT neurons (Ruggiero et al., 1999) and GABA neurons (Roche et al., 2003). Therefore, in addition to 5-HT innervation of the CeA by the dRN, the presence of reciprocal CRF fibers projecting from the CeA to the dRN indicates a complex interplay between these brain regions during stress.

Similarly, AVP is produced in regions of the amygdala and extended amygdala, such as medial amygdala (MeA) and bed nucleus of the stria terminalis (BNST), as well as hypothalamus, and innervates the CeA as well as the dRN (Rood and Beck, 2014; Hernandez et al., 2016). *Via* these connections and V_1*A*_ receptors, AVP has been demonstrated to indirectly influence 5-HT activity as well as anxiety and stress coping (Rood and Beck, 2014; Hernandez et al., 2016). Importantly, arginine vasopressin (AVP) potentiates the effects of CRF in the hypophysis (Liu et al., 1990; Oki et al., 1990) and amygdala (Elkabir et al., 1990). While active in the CeA (Winnicka, 1996; Veinante and Freund-Mercier, 1997; Ahn et al., 2001; Huber et al., 2005; Bosch and Neumann, 2010), and extended amygdala, AVP modulates neural responses to emotional or stressful stimuli (Ebner et al., 2002; Bosch and Neumann, 2010; Brunnlieb et al., 2013).

Low doses (0.1 to 1.0 μg) of icv CRF decrease 5-HT release in the terminal fields of the lateral septum and striatum, as well as discharge rates of dRN neurons *in vivo* (Price et al., 1998; Price and Lucki, 2001). The effects of CRF on 5-HT release in the lateral septum mimic reductions in lateral septum 5-HT release induced by swimming stress, an effect blocked by administration of a CRF receptor antagonist (Price et al., 2002). Additionally, stress-induced increases in serotonin turnover are further elevated by the administration of the CRF antagonist, α-helical CRF_9–41_, in a variety of brain regions including the amygdala (Li et al., 1998). These findings imply that, generally, CRF has an inhibitory effect on central 5-HT activity. However, increased 5-HT release in the CeA in response to restraint stress is inhibited by a CRF_1/2_ receptor antagonist [D-Phe-CRF_(12–41)_] delivered icv, suggesting a role for CRF in mediating stress-induced increases in 5-HT release (Mo et al., 2008). What is more, a higher dose (3.0 μg) of icv CRF has no effect on 5-HT release in the lateral septum but increases 5-HT release in the striatum while neuronal activity in some dRN neurons and increasing neuronal activity in others (Price et al., 1998; Price and Lucki, 2001). In addition, a small subpopulation of serotonergic neurons in the dRN have been shown to be excited by CRF *ex vivo*, (Lowry et al., 2000). This subpopulation of serotonergic dRN neurons is located in a subregion of the dRN containing neurons that project to the CeA and are topographically distinct from those that project to the striatum and lateral septum (Imai et al., 1986a,b). Lowry et al. (2000) thus hypothesized that 5-HT neurons within the dRN may be inhibited or activated by CRF based on topography.

Behavioral and clinical studies implicate central CRF and 5-HT in the pathophysiology of drug relapse, anxiety disorders, trauma- and stressor-related disorders such as posttraumatic stress disorder (PTSD), depression, and other psychiatric disorders exacerbated by stress (Chalmers et al., 1996; Coplan et al., 1996; Deakin, 1998; Holsboer, 1999; Keck and Holsboer, 2001; Le et al., 2002; Waters et al., 2015). A common feature of these disorders is that previous stress experience may promote inappropriate stress responsiveness (Herman and Cullinan, 1997; Chaouloff et al., 1999; Kosten and Ambrosio, 2002). In rat models, the effects of prior stress on HPA axis and behavioral responses to subsequent stressors (Bhatnagar and Dallman, 1998; Chung et al., 2000) appear to be mediated through central CRF and 5-HT systems. For example, prior stress enhances c-fos expression and CRF mRNA levels within the CeA, as well as CRF receptor binding sites in the dRN, and *Tph*_2_ mRNA expression in the dRN, in response to a novel stressor (Ladd et al., 1996; Albeck et al., 1997; Bhatnagar and Dallman, 1998; Chung et al., 2000; Donner et al., 2018). Related findings show that excitatory responses of dRN neurons to locally applied CRF are enhanced by prior restraint stress (Lowry et al., 2000); and that prior stress increases CRF-evoked stress behavior (Pelton et al., 1997) as well as stress-induced terminal 5-HT release (Jordan et al., 1994). Importantly, depletion of central 5-HT abolishes elevated behavioral responses to social stress in pre-stressed rats (Chung et al., 2000), and administration of a CRF receptor antagonist blocks the development of stress-induced behavioral sensitization to amphetamine (Cole et al., 1990). The experiments presented here, begin to unravel relationships in this system, by testing these *a priori* hypotheses: (1) The release of 5-HT in the CeA will not be modified by low dose rat/human CRF (rhCRF), or AVP alone. As CRF terminals and CRF_1_ receptors in the dRN are located primarily on GABA neurons that inhibit 5-HT release in response to stress (Roche et al., 2003), we hypothesize, (2): that subjecting rats to prior exposure to restraint stress will reduce extracellular 5-HT in CeA after CRF treatment (icv). This is despite the fact that icv administration of CRF will also result in binding of CRF receptors in the CeA, and potentially stimulate local neuronal activity (Mo et al., 2008; Skorzewska et al., 2009). Although the direct effect of icv CRF may be to decrease GABA concentration in the CeA (Skorzewska et al., 2009), some of those GABA neurons belong to disinhibitory circuitry within the CeA. The result may be to yield enhanced GABAergic inhibition of CRF-induced 5-HT release in the CeA following prior stress. Finally, since AVP potentiates the effects of CRF (Elkabir et al., 1990; Liu et al., 1990), and are active in the CeA (Winnicka, 1996; Veinante and Freund-Mercier, 1997; Ahn et al., 2001; Huber et al., 2005; Bosch and Neumann, 2010) to modulate neural responses to emotional or stressful stimuli in the amygdala (Ebner et al., 2002; Bosch and Neumann, 2010; Brunnlieb et al., 2013), we hypothesize that, 3. Similar to the effects of prior stress, AVP will act synergistically with CRF (Shabanov and Lebedev, 2008) to inhibit 5-HT release in the CeA.

## Materials and methods

### Subjects and housing

Male rats (280–500 g; Harlan Sprague-Dawley; Indianapolis, IN, USA) from a local breeding colony were housed as a group with a reversed 12:12 light:dark cycle (lights off from 6:00–18:00) with free access to food and water. All surgical and behavioral procedures were performed in a manner that minimized suffering and the number of animals used was in accordance with the National Institutes of Health’s *Guide for the Care and Use of Laboratory Animals* (NIH Publications No. 80-23), USDA (National Research Council) and Society for Neuroscience Guidelines and approved by the Institutional Animal Care and Use Committee of the University of South Dakota.

### Experimental design

**FIGURE 1:**
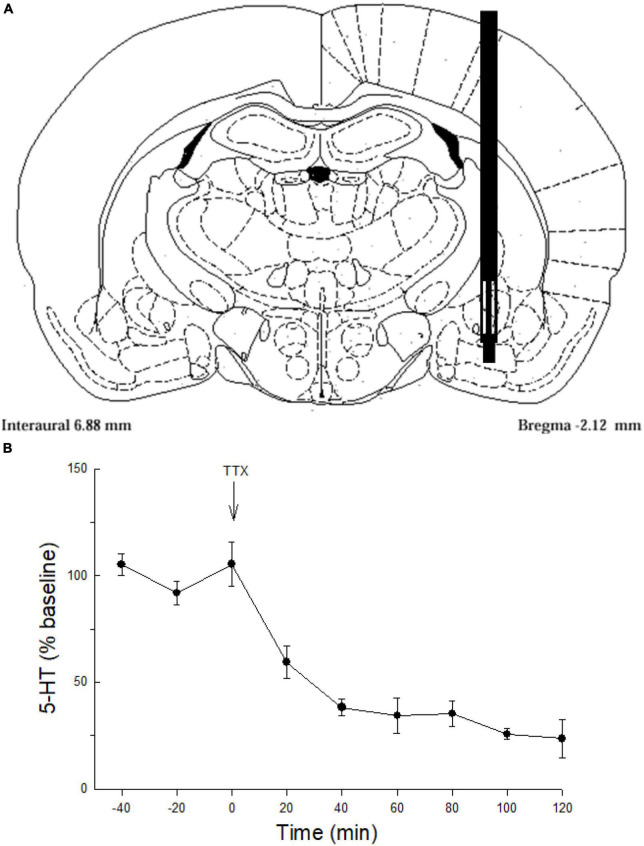
Serotonin sampling and neuronal origin in the CeA. **(A)** Representative schematic of microdialysis probe tip placement within the central nucleus of the amygdala (Paxinos and Watson, 1998). **(B)** Serotonin (5-HT) release (mean ± SEM; *N* = 4 rats) in the CeA over time. Values for –40, –20, and 0 min were used to normalize the data; presented as percent of baseline. Tetrodotoxin (TTX) perfused though the microdialysis probe significantly attenuates serotonin (5-HT) release in the CeA compared with samples taken prior to TTX treatment (–40, –20, 0 min; GLM-RMANOVA; *p* < 0.05).

Testing for synergistic relationships between prior stress, AVP, and CRF on 5-HT release in the CeA required 4 experiments, with microdialysis collection lasting typically for 4 h (Figure 1) and immediate HPLC measurement of 5-HT at each 20 min sampling point. In a few cases, where dialysis probes did not remain patent, limiting viable sample sizes, our sampling period varied slightly. First, the TTX experiment was only extended for 2 h. In prior stress experiments delivering 3 μg of CRF (220 min) sampling was only limited by one 20 min time period, and CRF + AVP treatments were extended to 260 min.

### Neuronal release of 5-HT

To validate neuronal sources of 5-HT release the Na^+^ channel blocker tetrodotoxin (Sigma Chemical Co., Saint Louis, MO, USA) was delivered (TTX, 25 ng/μl, *N* = 4; Figure 1B) in artificial cerebrospinal fluid (aCSF) *via* the microdialysis probe (Figure 1A). Inhibition of action potentials with TTX markedly reduced 5-HT overflow indicating that most of the 5-HT was derived from neuronal release (Figure 1B).

### CRF, prior stress, and CeA 5-HT release

To ascertain the effects of CRF alone, and combined with prior stress, on extracellular 5-HT release in the CeA, animals were randomly assigned to one of two stress treatments: No Restraint Stress (NS), or Restraint Stress (S), and one of three icv CRF treatments: 0 μg (saline control), 3 μg, or 10 μg CRF. Stress exposure followed 24 h later by icv CRF treatment resulted in 6 groups: 1. saline alone (*N* = 4), 2. 3 μg CRF alone (*N* = 4), 3. 10 μg CRF alone (*N* = 4), 4. Restraint Stress followed by saline (*N* = 4), 5. Stress plus 3 μg CRF (*N* = 6), 6. Stress prior to 10 μg CRF (*N* = 8).

### AVP, prior stress, and CeA 5-HT release

To examine the relationship between AVP, Stress, and 5-HT release in CeA, rats were randomly assigned to one of two stress treatments: No Restraint Stress (NS), or Restraint Stress (S), and one of two icv AVP treatments: 0 μg (saline control), 3 μg CRF. Stress exposure followed 24 h later by icv AVP treatment resulted in 4 groups: 1. saline alone (*N* = 7), 2. AVP alone (3 μg; *N* = 7), 3. Restraint Stress followed by saline (*N* = 7), 4. Stress prior to 3 μg CRF (*N* = 8).

### Synergistic effects of AVP + CRF

Finally, to assess a potential synergistic effect of AVP on CRF influence in CeA 5-HT release in the absence of prior restraint stress, two additional group were added. These groups were treated with 1. Saline (*N* = 7), or 2. AVP (3 μg) + CRF (10 μg) (*N* = 8), but was not restrained prior to the icv saline, or icv AVP and CRF treatments.

### Surgery, cannulae, and probe placements

Rats were anesthetized (ketamine-xylazine; 100 mg and 10 mg/kg respectively) for placement of guide cannulae (Plastics One, Roanoke, VA, USA). One cannula was used to guide delivery of icv saline, CRF, or AVP. A second cannula was implanted contralaterally and was directed toward the CeA for microdialysis. For icv drug administration, the guide cannula (26 ga cut to 1.5 mm) was inserted above the left or right lateral ventricle [from bregma; AP: −1.0 and ML: 1.5; (Paxinos and Watson, 1998)]. For microdialysis probe placement, the cannula was aimed at the CeA (Figure 1; from bregma in mm: AP = −2.0, ML = 4.1, DV = −7.0) in the opposite hemisphere. Animals recovered in individual cages for 3 days before experimental procedures.

### Prior stress

Restraint Stress consisted of placing an animal for 1 h in a 6.4 cm diameter PVC tube with several 1.3-cm ventilation holes. Rats freely entered the tube, with a screen mesh preventing further forward movement. They could not turn around. A rubber stopper was inserted to prevent exiting by backing out. Rats were restrained after 4 h of darkness, during the dark phase (scotophase) of the photoperiod (between 1100 and 1400 h) in a darkened room adjacent to animal housing quarters approximately 24 h prior to treatment and collection of microdialysis samples.

### Neuropeptide administration

All of the CRF used in these experiments is of the sequence secreted by rats and humans (rhCRF; Sigma-Aldrich, St. Louis, MO, USA; Cat. No. C-3042). The neuropeptides CRF and AVP (Sigma-Aldrich, acetate salt; Cat. No. V-9879) were dissolved in sterile 0.9% saline to produce dosages (per μl) 3 μg (CRF, AVP), and 10 μg (CRF). All icv injections (1 μl) were delivered over the course of 1 minute into the lateral ventricle using a Hamilton 5 μl syringe. Injections were made after reaching a stable baseline (<10% variation of 5-HT signal for 3 consecutive microdialysis samples), followed by microdialysis sampling at 20 min intervals for approximately 4 h.

### *In vivo* microdialysis

On the morning of dialysis, rats were briefly anesthetized with isoflurane and a custom concentric dialysis probe (Hoffman et al., 2002) exposed cellulose tip length of approximately 2.0 mm (M.W. cut-off 5000, Travenol Laboratories, Deerfield, IL, USA) was implanted in the CeA. Probes were connected to a liquid swivel (Instech Laboratories, Plymouth Meeting, PA, USA) that enabled the rats to move freely in a 38-l terrarium. Terraria walls were covered, and illumination was provided by using 25 W red lights. A modified Ringer’s solution (in mM: 137 NaCl, 1.2 CaCl_2_, 1.2 MgCl_2_, 2.4 KCl, 0.9 NaH_2_PO_4_, 1.4 Na_2_HPO_4_; (Moghaddam and Bunney, 1989) was perfused through the probe at a flow rate of 0.4 μl/min employing a CMA/100 microinjection pump (CMA, North Chelmsford, MA, USA). The outlet line emptied into a microcentrifuge vial attached above the liquid swivel, which allowed for samples to be collected with minimal disturbance of the rats. Probe recovery rates for 5-HT, determined in modified Ringer’s at a flow rate of 0.4 μl/min, varied between 10–20%. After a 4–6 h washout period samples were collected and analyzed until there was a stable 5-HT baseline (three samples with < 10% variation).

### HPLC

Dialysate samples were analyzed for 5-HT during experiments, immediately following collection at 20 min intervals. Samples were manually injected into a Rheodyne 7125 injector (Bioanalytical Systems, BAS, West Lafayette, IN, USA) with a 5 μl sample loop. A custom-built pneumatic nitrogen displacement pump [(Bradberry et al., 1991, 1993), 2000 psi] was connected to an LC-4B electrochemical detector (BAS) and a thin-layer glassy carbon electrode (BAS) set at + 0.55 V with respect to an Ag/AgCl_2_ reference electrode. Separation was accomplished using a 100 mm, 3 μm reverse-phase Sepstick C_18_ column (BAS). The mobile phase was optimized for 5-HT separation (4.7 g NaH_2_PO_4_, 150 mg EDTA, 120 μl triethylamine, 433 mg octanesulfonic acid, and 115 ml acetonitrile in 1 l H_2_O, with a final pH 5.45–5.55). Serotonin peaks in the samples were identified by comparison with 5-HT standards injected prior to each experiment. The retention time for the 5-HT peak was approximately 15 min. At the end of the experiments, rats were administered a lethal dose of chloral hydrate anesthesia. The brains were removed and preserved in 10% phosphate-buffered formalin (Fisher Scientific, Kalamazoo, MI). Brains were sliced at 50 μm using a Leica 1850 cryostat (Leica Instruments, Heidelberg, Germany) and stained with cresyl violet to verify the probe and cannulae implant sites.

### Data analysis

Peak heights were measured, and baseline values monitored for three consecutive pretreatment peaks. All baseline values for samples used varied no more than 10% across all samples, and the mean of those values for 5-HT were used to normalize all results to be presented as a percentage of baseline. Data were analyzed by a General Linear Model Two-Way ANOVA with repeated measures (GLM-TWRMANOVA; using both Sigma Stat and SPSS software) with Bonferroni and Dunnett’s *post hoc* tests where appropriate. For all analyses, we examine the data relative to specific assumptions for parametric statistics [such as Homogeneity of variance (similar homoscedasticity)], which may be modified by the number of sampling times. However, due to dialysis probe clogging, the 240-min time point is missing for 3 μg CRF treatment group. To address this problem, we used two techniques, (1) run the entire ANOVA using the largest number of sampling times found for all groups. In addition, (2) when the sample size is relatively large, as is the case with 15 versus 14-time sampling points, the inequality of variances moves toward zero, and the outcome of the ANOVA is robust, in spite of the missing value. Thus, we were able to run the full ANOVA, with consistent results.

## Results

Dialysate samples collected from the CeA (Figure 1A) demonstrated that TTX blockade of Na^+^channels in the CeA significantly decreased extracellular 5-HT (Figure 1B) indicating that 5-HT overflow measured in these experiments comes primarily from neuronal release.

**FIGURE 2:**
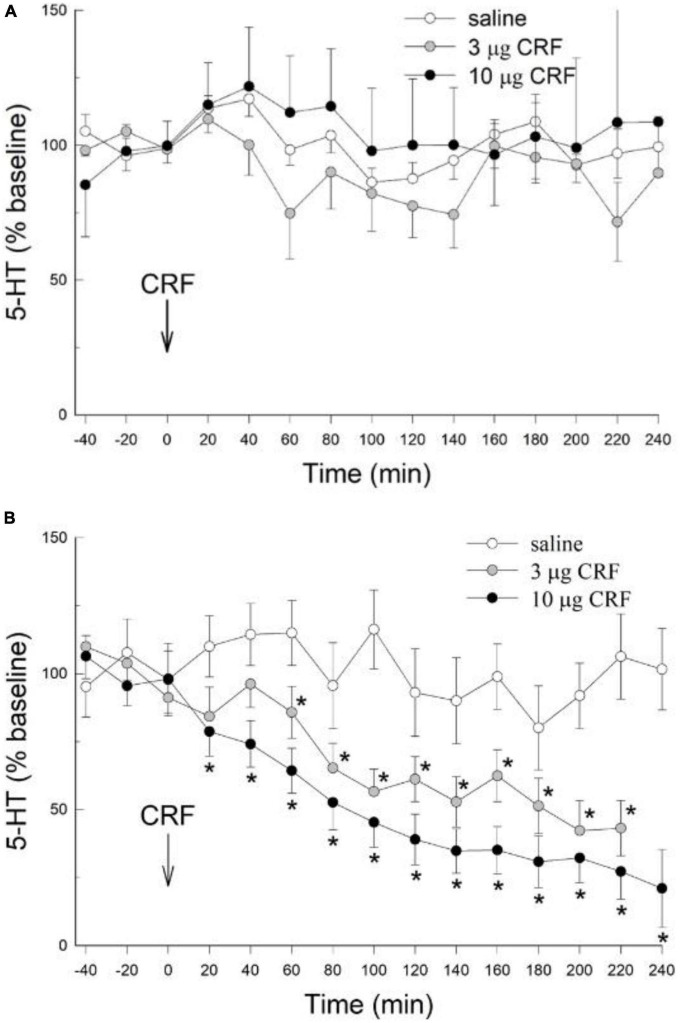
Prior stress is necessary to promote icv CRF reduction of 5-HT in the CeA. **(A)** In naïve, unstressed rats, treated icv with saline (*N* = 4), or rat/human CRF (CRF) treatment (3 or 10 μg) have no significant (no *) effect on the release of serotonin (5-HT; mean ± SEM) in the CeA over time (data are normalized to the 3 samples prior to CRF treatment at 0 min, arrow). The CRF groups were not statistically different from the saline group (condition: *p* > 0.29, condition x time: *p* > 0.35; GLM-TWRMANOVA). **(B)** In stressed rats (1 h of restraint stress 24 h prior to infusion), icv CRF significantly [for 3 (*N* = 6) and 10 μg (*N* = 8)] reduces 5-HT release in the CeA of rats, compared with CRF alone [*GLM-TWRMANOVA, condition, *p* < 0.0018 panel **(A)**], samples taken prior to CRF treatment (–40, –20, 0 min; time, *p < 0.001), or saline controls (*N* = 4; condition x time, *p < 0.001) that also received prior stress. In animals exposed to prior stress, 5-HT responses in the CeA to the 3 μg and 10 μg doses of CRF did not differ significantly.

There were no significant effects of either the 3 or 10 μg doses of icv CRF on extracellular 5-HT in the CeA (condition: *F*_2,146_ = 1.36; *p* > 0.287; condition x time: *F*_28,146_ = 1.1 *p* > 0.35; Figure 2A) when compared to the saline vehicle; in rats that were not restrained prior to the experiment. While there was a significant time effect (time: *F*_14,146_ = 1.89, *p* < 0.031), there were no significant differences between dosage treatment groups at any individual time point (*p* > 0.05, Dunnett’s *post hoc* test). These results in unstressed animals were significantly different from those treated with restraint, as rats exposed to restraint stress 24 h prior to administration of CRF exhibited pronounced and significant decreases in extracellular 5-HT (condition: *F*_2,145_ = 15.091; *p* < 0.0018) which persisted for the duration (time; *F*_14,145_ = 15.096; *p* < 0.001; condition x time interaction: *F*_28,145_ = 2.918; *p* < 0.001) of the experiment (Figure 2B). In contrast to rats without prior restraint stress, for which no effect of CRF on CeA extracellular 5-HT was evident (Figure 2A), both the 3 μg and 10 μg dose of CRF decreased extracellular 5-HT in the CeA after restraint stress, compared to samples taken prior to CRF treatment (−40, −20, 0 min = time effect), or to saline controls that also received prior stress (Figure 2B). The serotonergic responses in the CeA to the 3 μg and 10 μg doses of CRF did not differ significantly in animals exposed to prior stress.

**FIGURE 3:**
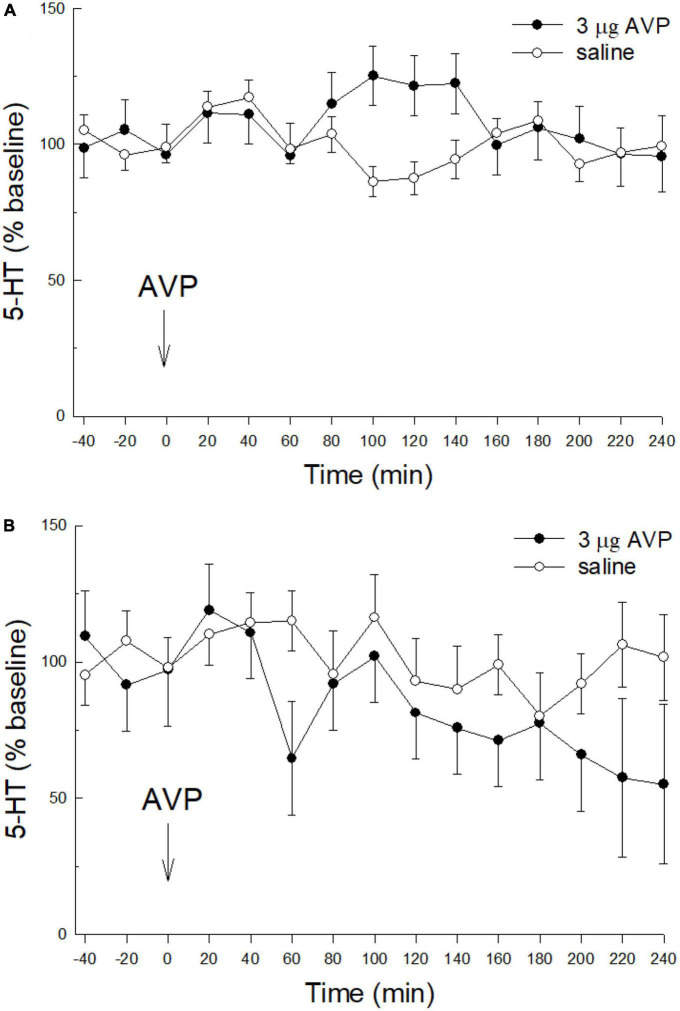
Prior stress is necessary to promote icv AVP reduction of 5-HT in the CeA. **(A)** Similar to CRF, in naïve, unstressed rats, treated icv with saline (*N* = 7), or 3.0 μg vasopressin (AVP, *N* = 7) alone do not affect the release of 5-HT in the CeA (condition: *p* > 0.39; time: *p* > 0.6; condition x time: *p* > 0.77; GLM-TWRMANOVA). **(B)** The combination of stress prior to icv AVP delivery (*N* = 8) produces a significant decline in CeA 5-HT release, compared with restraint stress followed by saline (*N* = 7; time: *p* ≤ 0.0343; GLM-TWRMANOVA).

Similar to CRF, icv injection of AVP (3 μg, icv) alone with no prior stress had no effect on extracellular serotonin in the CeA (Figure 3A; condition; *F*_1,150_ = 0.776; *p* > 0.394: time; *F*_14,150_ = 0.86; *p* > 0.604: condition x time; *F*_14,150_ = 1.628; *p* > 0.776). Although CRF nor AVP alone or without restraint stress influenced extracellular 5-HT in the CeA, when 60 min of restraint stress was applied 24 h prior to AVP (3 μg, icv; Figure 3B) administration, similar to icv CRF plus prior stress, there was a significant decrease in extracellular 5-HT over time (condition: *F*_1,27_ = 2.64, *p* > 0.2; time: *F*_14,27_ = 2.25; *p* ≤ 0.0343; condition x time: *F*_14,27_ = 1.24; *p* > 0.3024; Figure 3B).

**FIGURE 4:**
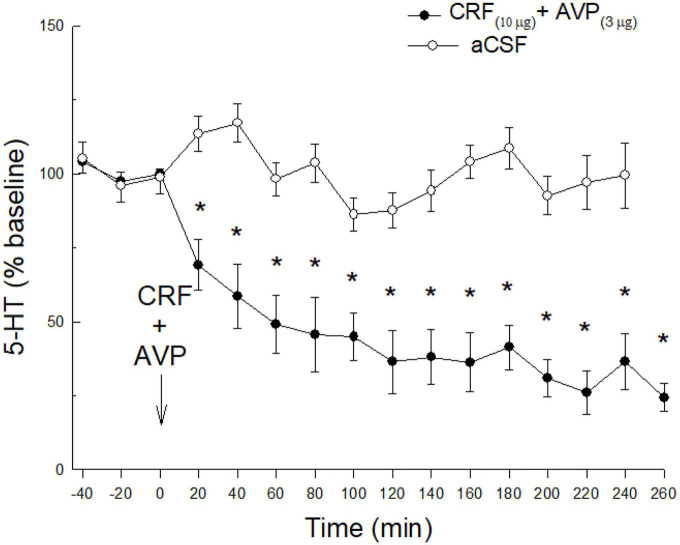
Without stress, a combination of icv CRF and AVP, is necessary to promote reduction of 5-HT in the CeA. While either CRF or AVP combined with prior stress (Figures 2B, 3B) produce significant reductions of 5-HT release in CeA, in the absence of prior stress, icv AVP plus CRF (*N* = 8) produce synergistic effects resulting in a dramatic and persistent decline in extracellular 5-HT in the CeA (*time: < 0.0001; condition x time: *p* < 0.0001). This decline is significant compared with samples taken prior to CRF treatment (time: –40, –20, 0 min), or saline controls (*N* = 7; condition x time) that also received no prior stress.

Although neither CRF nor AVP had any effect in the absence of restraint stress, co-administration of CRF (10 μg) and AVP (3 μg) substantially decreased 5-HT release (time: *F*_15,167_ = 10.3 *p* < 0.0001; condition x time: *F*_15,167_ = 5.25; *p* < 0.0001; Figure 4) in unstressed animals. Similar to the effect of prior stress, co-administered CRF and AVP produced a prolonged reduction in 5-HT release in the CeA.

## Discussion

Administration of CRF (icv) inhibited release of 5-HT in the CeA (as measured by microdialysis) in freely moving rats, but only if those rats had been exposed to 1 h of restraint stress 24 h prior to administration of CRF. Similarly, AVP (icv) alone did not modify 5-HT release in the CeA, but did so following restraint stress. Thus, release of 5-HT in the CeA is not acutely modified by CRF or AVP, in the absence of some modulating condition or factor (Mo et al., 2008). Interestingly, similar to prior stress, AVP in combination with CRF resulted in a conspicuous inhibition of 5-HT release in the CeA. In this case, by including prior stress or AVP, CRF inhibited 5-HT release as much as TTX and for up to 240 min. These results are consistent with previous reports demonstrating a primarily inhibitory effect of CRF on presumably dRN derived serotonin release (Li et al., 1998; Price et al., 1998; Kirby et al., 2000; Roche et al., 2003) and a sensitization of CRF-mediated systems by exposure to prior stress (Curtis et al., 1995; Pelton et al., 1997; Price et al., 1998; Kirby et al., 2000, 2008). However, a specific small subset of serotonergic neurons in the dRN is stimulated by CRF (Lowry et al., 2000). These results also demonstrate that stress and neuropeptide action functionally alter CRF-mediated effects on 5-HT release in the CeA (Mo et al., 2008).

The experiments presented were designed to give an indication of the complex relationships between stress, CRF, and AVP on neuronally driven 5-HT output (Summers et al., 2003) in the CeA, to lay a framework for which additional studies would consider the specific actions of CRF_1_ and CRF_2_ receptor agonists and antagonists (Ronan and Summers, 2011), V_1A_ agonists and antagonists, the anatomical specificity of their actions through intracranial (intra-CeA) delivery and genetic manipulation, as well as the type and timing of stressors and/or pre-stressors. The effect on serotonergic neurons appears to be also affected through CRF/GABA interactions in the dorsal raphé (Summers et al., 2003), and while both CRF_1_ and CRF_2_ receptors diminish serotonergic response there (Kirby et al., 2008), it is not clear whether this is also true relative to prior stress and/or AVP actions in a downstream target like the CeA. Future experiments are necessary to divulge these complex relationships.

Our results demonstrate potential effects of CRF in CeA, although because it was delivered icv, we cannot discriminate whether the effects are direct or circuitous. An underappreciated factor, which may be important for this work, is that neuropeptides may be actively released into CSF as a mechanism of transport (Vigh et al., 2004). As such, icv administration of CRF may be particularly physiologically relevant and mimic an endogenous route for CRF signaling. For CRF, ventricular volume flow results in stress related physiological responses and more rapid clearance than simple bulk flow, suggesting specificity of function for this type of transport (Rock et al., 1984; Oldfield et al., 1985). It is important to note that anatomically, many critical CRF or urocortin-producing and CRF-responsive brain regions are situated paraventricularly (Bittencourt et al., 1999; Bittencourt and Sawchenko, 2000).

Having made a case for volume signaling, the results also suggest a potentially direct stimulation by CRF on CeA neurons or terminals (Rainnie et al., 1992) received from other brain regions such as the dRN (Vertes, 1991; Hale et al., 2008; Johnson et al., 2015). While the effects of CRF on 5-HT terminal activity in the CeA have not been directly explored, icv administration of a CRF receptor antagonist dramatically increases the local concentration of GABA in the CeA (Skorzewska et al., 2009), which could inhibit release from 5-HT terminals. Additionally, the CeA expresses low levels of CRF receptor binding sites (De Souza et al., 1985; Liebsch et al., 1995), and is innervated by CRF-positive axon terminals derived from the lateral hypothalamus, the dRN, and other amygdaloid nuclei, as well as CRF neurons derived from the CeA itself, (Uryu et al., 1992; Gray, 1993). Similarly, global antagonism of CRF receptors attenuates responses to stressors (Heinrichs et al., 1994; Aloisi et al., 1999; Koob and Heinrichs, 1999; Roche et al., 2003). Antagonism of CRF receptors in the CeA or dRN reduces behavioral responses to stressors (Swiergiel et al., 1993; Liebsch et al., 1995; Hammack et al., 2003; Skorzewska et al., 2009), while increasing intra-CeA concentrations of GABA (Skorzewska et al., 2009).

Similarly AVP, which can be produced in the hypothalamus or extended amygdala (Rood and Beck, 2014; Hernandez et al., 2016) has been demonstrated to have effects in dRN, but also in CeA *via* V_1A_ receptors (Bosch and Neumann, 2010; Rood and Beck, 2014; Hernandez et al., 2016). However, while V_1A_ activations indirectly excites serotonergic neurons in dRN, our results with prior stress + AVP or AVP + CRF demonstrate inhibition of serotonergic release in CeA. In CeA, V_1A_ receptors act on GABAergic neurons (Hernandez et al., 2016), presumably stimulating inhibitory GABA output, and producing anxious behavior. In another study, combined icv injections, as well as intracerebral injections of CRF with AVP into the CeA, act synergistically to potentiate or profoundly reduce behavior (Elkabir et al., 1990). The synergistic response of CRF + AVP in the pituitary relies on the V_1A_ activation of Phospholipase C/Protein Kinase C/Ca++/Calmodulin system, even though CRF acts through CRF_1_ receptors and cAMP (Liu et al., 1990; Oki et al., 1990). Since V_1A_ receptors enhance 5-HT signaling in dRN, and are found on GABAergic neurons in CeA, it suggests to us that the synergism of CRF and AVP to reduce 5-HT release in CeA likely is coupled to GABAergic inhibition. The complex GABAergic circuitry of the CeA likely plays an important role in modulating the limited effects of CRF and AVP alone, as well as the potentiated reduction of 5-HT release due to prior stress with CRF or AVP, and the combination of CRF and AVP. Additional experimentation will be necessary to determine the mechanisms involved.

The effect of prior restraint stress exposure, 24 h before icv delivery of CRF, was to produce inhibited 5-HT release in the CeA, not evident in the absence of the prior stressor. The results suggest that the prior exposure to stress modified function either in the raphé (Summers et al., 2003), or in CeA neurons influencing raphé terminals there. Prior exposure to various types of stressors alters other measures of CRF activity. Immobilization stress increases CRF and AVP release into the median eminence within 30 min, producing a rapid adrenocorticotropic hormone (ACTH) response, and elevated circulating levels of glucocorticoids, with the number of CRF plus AVP terminals increasing 2-fold over the next 9–16 days (de Goeij et al., 1991). Similarly, 90 min of immobilization stress enhanced the expression of mRNA for CRF, as well as CRF_1_ receptors, in the hypothalamus (Rivest et al., 1995). Prior stress (footshock) sensitizes the excitatory effects of subsequent CRF on the acoustic startle reflex in rats (Pelton et al., 1997). Furthermore, prior exposure of rats to 30-min of mild footshock significantly attenuates CRF-induced firing of locus ceruleus (LC) neurons (Curtis et al., 1995). Subordinates in the chronic social stress visible burrow model show increased CRF mRNA in the CeA. Non-responsive subordinates that have an impaired corticosterone response to a novel restraint stressor also have decreased mRNA for CRF in the PVN (Albeck et al., 1997). Together the results suggest that prior stress may modify the functional responses of numerous stress-related brain regions, and that our results may have more than one cause.

With icv delivery of CRF in the absence of prior stress, there was no change in 5-HT release in the CeA. Serotonergic and CRF systems are both activated during stressful events. Serotonin may be regulated by, and in turn, may regulate central CRF (Marcinkiewcz et al., 2016). The PVN has a robust serotonergic innervation [for review, see (Chaouloff, 1993)], which stimulates CRF secretion into the median eminence of the hypothalamus *in vivo* (Gibbs and Vale, 1983) as well as *in vitro* (Holmes et al., 1982; Nakagami et al., 1986; Calogero et al., 1989). Acute and chronic administration of the 5-HT_1A_ receptor agonists, 8-OH-DPAT or ipsapirone, increase plasma concentrations of corticosterone whereas only acute administration increases plasma ACTH. Chronic administration of these agonists also causes increased CRF concentrations in the hippocampus and piriform cortex; however, only 8-OH-DPAT causes CRF increases in the amygdala and entorhinal cortex (Owens et al., 1990). Thus, activation of 5-HT_1A_ receptors can influence CRF neurons in extrahypothalamic areas. Optogenetic studies have demonstrated a positive effect of 5-HT action on CRF neurons in the bed nucleus of the stria terminalis (BNST), which further enhances anxiety and aversive learning (Marcinkiewcz et al., 2016), demonstrating the possibility of 5-HT effects on extended amygdala CRF. It is possible that a delayed negative feedback loop exists in which 5-HT can activate CRF systems but, as the present experiment suggests, once activated, CRF may inhibit its own release by inhibiting further 5-HT release. Thus, the reduction in CeA 5-HT release in response to prior stress and subsequent icv CRF treatment, may play a role in regulating CRF produced in the CeA, or simply influence GABAergic neurons there. The effect of icv CRF on 5-HT release appears to be specific to brain regions known to innervate the CeA, such as the dRN and mRN, but may also affect other brain regions such as BNST, PFC, or lateral habenula which also innervate the dRN, mRN, or CeA and are also involved regulating stress and anxiety (Challis et al., 2014; Ohmura et al., 2014; Dolzani et al., 2016; Marcinkiewcz et al., 2016).

Serotonin in the CeA plays an important role in anxiety and activation of the HPA axis. Microinjection of the 5-HT_3_ agonist in the amygdala has both anxiogenic and anxiolytic effects depending on the measure (Gargiulo et al., 1996). Stressor induced release of CRF is inhibited in rats with depleted amygdalar 5-HT by local administration of 5,7-dihydroxytryptamine, though hypothalamic 5-HT content was not affected (Feldman and Weidenfeld, 1995). Local pretreatment with ketanserin (a 5-HT_2_ receptor antagonist) in the amygdala has the same inhibitory effect on HPA axis responses (Feldman and Weidenfeld, 1998; Feldman et al., 1998). This suggests that the decreased 5-HT in the CeA seen in this experiment is part of a negative feedback circuit regulating HPA axis function, which may have downstream effects on anxious behavior and affect. Following a prior stressor, CRF-induced decreases of 5-HT release in the CeA could attenuate subsequent release of CRF from the hypothalamus. This provides a mechanism by which extrahypothalamic CRF can modulate the overall stress response of an organism.

Inhibition of 5-HT release in the CeA may be mediated by CRF-induced increases of GABA release. Local GABA fibers, terminals and cell bodies are located in the CeA (McDonald, 1985; Ottersen et al., 1986). In certain brain regions, it has been suggested that CRF synthesis and release may be regulated by GABA (Tran et al., 1999). Some evidence indicates that CRF stimulates GABA release in other brain regions. For example, icv administration of CRF causes increases in GABA in both the globus pallidus and caudate nucleus (Sirinathsinghji and Heavens, 1989). Thus, it is possible that CRF increases GABA in the CeA, which, in turn, inhibits the release of 5-HT. In the hypothalamus, the GABA_A_ receptor antagonist bicuculline induces pronounced increases in 5-HT release (Luine et al., 1999). Behavioral studies support this notion. Behavioral effects of exogenously administered CRF, consistent with an increase in anxiety, can be reversed by chlordiazepoxide, a classic anxiolytic drug that acts on the benzodiazepine allosteric site on the GABA_A_ receptor (Britton et al., 1985). Intercalated amygdala neurons, which use GABA as their neurotransmitter and project primarily to the CeA, were found to spontaneously fire at much higher rates in response to CRF than commonly observed in neurons within neighboring amygdaloid nuclei (Collins and Pare, 1999). Iontophoretic application of the GABA_A_ antagonist bicuculline, increased firing rates of 20% of CeA cells (Veinante and Freund-Mercier, 1998). Taken together, these results suggest that CeA neurons are under tonic GABAergic inhibitory control and is consistent with behavioral results that indicate that GABA in the amygdala tonically inhibits anxious behavior while 5-HT has been suggested to facilitate it (Graeff, 1994). It is also plausible that 5-HT, with its primarily inhibitory action on cells (Jacobs and Azmitia, 1992), provides a tonic inhibitory control of GABAergic neurons in the CeA. Decreases in 5-HT, as seen in this work, would then disinhibit GABAergic inhibition of CeA neurons causing, in effect, an excitatory action. More work needs to be done to understand the precise role of GABA in the regulation of CRF and 5-HT activity in the CeA.

In conclusion, the response of 5-HT release in CeA in response to icv CRF or AVP due to prior stress seen in this experiment may be an important mechanism regulating central and HPA stress responses. These synergistic effects are mirrored by a combination of CRF plus AVP treatment. Prior experience changes CRF-mediated mechanisms in the brain. Synergistic CRF plus AVP actions on 5-HT release may help explain the mechanism of prior stress modulation. This may be an etiological factor underlying stress-induced psychopathology.

## Data availability statement

The raw data supporting the conclusions of this article will be made available by the authors, without undue reservation.

## Ethics statement

The animal study was reviewed and approved by the University of South Dakota IACUC.

## Author contributions

PR: conceptualization, methodology, validation, formal analysis, investigation, writing—original draft, writing—review and editing, and visualization. WK: methodology, validation, formal analysis, investigation, and writing—review and editing. PJ: methodology, validation, formal analysis, investigation, and writing—review and editing. CL: conceptualization, methodology, validation, and writing—review and editing. KR: methodology, validation, formal analysis, writing—review and editing, supervision, project administration, and funding acquisition. CS: conceptualization, methodology, validation, formal analysis, investigation, writing—original draft, writing—review and editing, visualization, supervision, project administration, and funding acquisition. All authors approved the final version for publication.
